# Supplementary material for: Pyruvate Dehydrogenase Kinase 1 inhibition mediated oxidative phosphorylation enhancement in cartilage promotes osteoarthritis progression
Source: BMC Musculoskelet Disord. 2023 Jul 20;24:597. doi: 10.1186/s12891-023-06585-6 (PMC10357736; doi:10.1186/s12891-023-06585-6)
Supplement: Supplementary file 1 — Additional file 1. [file 12891_2023_6585_MOESM1_ESM.zip › BP.Gsea.1653623667859/GOBP_BRANCHING_INVOLVED_IN_BLOOD_VESSEL_MORPHOGENESIS.html]

Details for gene set GOBP\_BRANCHING\_INVOLVED\_IN\_BLOOD\_VESSEL\_MORPHOGENESIS[GSEA]

|  || Dataset | 55457\_collapsed\_to\_symbols.cls.cls #OA\_versus\_normal.cls.cls #OA\_versus\_normal\_repos |
| Phenotype | cls.cls#OA\_versus\_normal\_repos |
| Upregulated in class | normal |
| GeneSet | GOBP\_BRANCHING\_INVOLVED\_IN\_BLOOD\_VESSEL\_MORPHOGENESIS |
| Enrichment Score (ES) | -0.57687205 |
| Normalized Enrichment Score (NES) | -1.9360192 |
| Nominal p-value | 0.0017391305 |
| FDR q-value | 0.04369172 |
| FWER p-Value | 0.538 |
Table: GSEA Results Summary

  

Fig 1: Enrichment plot: GOBP\_BRANCHING\_INVOLVED\_IN\_BLOOD\_VESSEL\_MORPHOGENESIS      
 Profile of the Running ES Score & Positions of GeneSet Members on the Rank Ordered List

  

| SYMBOL | TITLE | RANK IN GENE LIST | RANK METRIC SCORE | RUNNING ES | CORE ENRICHMENT || 1 | GBX2 | gastrulation brain homeobox 2 [Source:HGNC Symbol;Acc:HGNC:4186] | 1861 | 0.378 | -0.1107 | No |
| 2 | NRP1 | neuropilin 1 [Source:HGNC Symbol;Acc:HGNC:8004] | 3704 | 0.185 | -0.2372 | No |
| 3 | ACVR1 | activin A receptor type 1 [Source:HGNC Symbol;Acc:HGNC:171] | 3898 | 0.165 | -0.2374 | No |
| 4 | FKBPL | FKBP prolyl isomerase like [Source:HGNC Symbol;Acc:HGNC:13949] | 4068 | 0.149 | -0.2371 | No |
| 5 | EDNRA | endothelin receptor type A [Source:HGNC Symbol;Acc:HGNC:3179] | 4663 | 0.099 | -0.2743 | No |
| 6 | FGF8 | fibroblast growth factor 8 [Source:HGNC Symbol;Acc:HGNC:3686] | 4761 | 0.090 | -0.2738 | No |
| 7 | SEMA3E | semaphorin 3E [Source:HGNC Symbol;Acc:HGNC:10727] | 4815 | 0.086 | -0.2702 | No |
| 8 | MDK | midkine [Source:HGNC Symbol;Acc:HGNC:6972] | 5828 | 0.007 | -0.3482 | No |
| 9 | ENG | endoglin [Source:HGNC Symbol;Acc:HGNC:3349] | 7213 | -0.097 | -0.4470 | No |
| 10 | LEF1 | lymphoid enhancer binding factor 1 [Source:HGNC Symbol;Acc:HGNC:6551] | 7467 | -0.116 | -0.4563 | No |
| 11 | TGFBR2 | transforming growth factor beta receptor 2 [Source:HGNC Symbol;Acc:HGNC:11773] | 8241 | -0.174 | -0.5007 | No |
| 12 | PLXND1 | plexin D1 [Source:HGNC Symbol;Acc:HGNC:9107] | 8438 | -0.189 | -0.4990 | No |
| 13 | SIRT6 | sirtuin 6 [Source:HGNC Symbol;Acc:HGNC:14934] | 8532 | -0.196 | -0.4886 | No |
| 14 | SHH | sonic hedgehog signaling molecule [Source:HGNC Symbol;Acc:HGNC:10848] | 8941 | -0.227 | -0.4999 | No |
| 15 | ABL1 | "ABL proto-oncogene 1, non-receptor tyrosine kinase [Source:HGNC Symbol;Acc:HGNC:76]" | 9081 | -0.238 | -0.4893 | No |
| 16 | GDF2 | growth differentiation factor 2 [Source:HGNC Symbol;Acc:HGNC:4217] | 9223 | -0.250 | -0.4778 | No |
| 17 | RBM15 | RNA binding motif protein 15 [Source:HGNC Symbol;Acc:HGNC:14959] | 10499 | -0.373 | -0.5434 | Yes |
| 18 | IHH | Indian hedgehog signaling molecule [Source:HGNC Symbol;Acc:HGNC:5956] | 10886 | -0.415 | -0.5361 | Yes |
| 19 | STK4 | serine/threonine kinase 4 [Source:HGNC Symbol;Acc:HGNC:11408] | 11074 | -0.441 | -0.5111 | Yes |
| 20 | EDN1 | endothelin 1 [Source:HGNC Symbol;Acc:HGNC:3176] | 11276 | -0.468 | -0.4847 | Yes |
| 21 | NFATC4 | nuclear factor of activated T cells 4 [Source:HGNC Symbol;Acc:HGNC:7778] | 11363 | -0.481 | -0.4482 | Yes |
| 22 | NOTCH4 | notch receptor 4 [Source:HGNC Symbol;Acc:HGNC:7884] | 11441 | -0.495 | -0.4098 | Yes |
| 23 | KDR | kinase insert domain receptor [Source:HGNC Symbol;Acc:HGNC:6307] | 11454 | -0.496 | -0.3661 | Yes |
| 24 | FOXC2 | forkhead box C2 [Source:HGNC Symbol;Acc:HGNC:3801] | 12218 | -0.650 | -0.3671 | Yes |
| 25 | SRF | serum response factor [Source:HGNC Symbol;Acc:HGNC:11291] | 12549 | -0.773 | -0.3233 | Yes |
| 26 | VEGFA | vascular endothelial growth factor A [Source:HGNC Symbol;Acc:HGNC:12680] | 12629 | -0.813 | -0.2565 | Yes |
| 27 | COL4A1 | collagen type IV alpha 1 chain [Source:HGNC Symbol;Acc:HGNC:2202] | 12703 | -0.866 | -0.1844 | Yes |
| 28 | GNA13 | G protein subunit alpha 13 [Source:HGNC Symbol;Acc:HGNC:4381] | 12862 | -1.103 | -0.0976 | Yes |
| 29 | CTNNB1 | catenin beta 1 [Source:HGNC Symbol;Acc:HGNC:2514] | 12866 | -1.119 | 0.0026 | Yes |
Table: GSEA details [plain text format]

  

Fig 2: GOBP\_BRANCHING\_INVOLVED\_IN\_BLOOD\_VESSEL\_MORPHOGENESIS      
 Blue-Pink O' Gram in the Space of the Analyzed GeneSet

  

Fig 3: GOBP\_BRANCHING\_INVOLVED\_IN\_BLOOD\_VESSEL\_MORPHOGENESIS: Random ES distribution      
 Gene set null distribution of ES for **GOBP\_BRANCHING\_INVOLVED\_IN\_BLOOD\_VESSEL\_MORPHOGENESIS**
